# Ten years malaria trend at Arjo-Didessa sugar development site and its vicinity, Southwest Ethiopia: a retrospective study

**DOI:** 10.1186/s12936-019-2777-z

**Published:** 2019-04-24

**Authors:** Dawit Hawaria, Hallelujah Getachew, Guofa Zhong, Assalif Demissew, Kasahun Habitamu, Beka Raya, Ming-Chieh Lee, Delenasaw Yewhalaw, Guiyun Yan

**Affiliations:** 1 Yirgalem Hospital Medical College, Yirgalem, Ethiopia; 2 Department of Medical Laboratory Technology, Arbaminch College of Health Sciences, Arbaminch, Ethiopia; 3 Program in Public Health, University of California at Irvine, Irvine, CA 92697 USA; 4 Department of Medical Laboratory Sciences, College of Medicine and Health Sciences, Ambo University, Ambo, Ethiopia; 5 Department of Medical Laboratory Sciences, College of Medicine and Health Science, Kotebe Metropolitan University, Addis Ababa, Ethiopia; 6 Department of Medical Laboratory Sciences, Institute of Health, Jimma University, Jimma, Ethiopia; 7 Tropical and Infectious Diseases Research Center (TIDRC), Jimma University, Jimma, Ethiopia

**Keywords:** *Plasmodium falciparum*, *P. vivax*, Age distribution, Sex distribution, Temporal trend, Climatic factors, Sugarcane farming, Ethiopia

## Abstract

**Background:**

The trend analysis of malaria data from health facilities is useful for understanding dynamics of malaria epidemiology and inform for future malaria control planning. Changes in clinical malaria characteristics, like gender and age distribution are good indicators of declining malaria transmission. This study was conducted to determine the malaria trend at Arjo-Didessa sugar development site and its vicinity, southwest Ethiopia, from 2008 to 2017.

**Methods:**

Monthly malaria confirmed case data from 2008 to 2017 was extracted from 11 health facilities based on clinical registers at Arjo sugar development site and its vicinity, southwest Ethiopia. Both positivity rate and malaria incidence rate were calculated. Changes in malaria parasite species and seasonality were analysed; age structure and gender distribution were compared between different study periods. Trend in malaria incidence and climatic impact were analysed and past LLIN and IRS campaigns were used as dynamics modifier.

**Results:**

Over a period of 10 years, 54,020 blood film were collected for malaria diagnosis in the health facilities at the area, of which 18,049 (33.4%) were confirmed malaria cases by both microscopically and RDT. *Plasmodium falciparum, Plasmodium vivax*, and mixed infection (*P. falciparum* and *P. vivax*) accounted for 8660 (48%), 7649 (42.4%), and 1740 (9.6%) of the malaria cases, respectively. The study also revealed that *P. vivax* was the predominant over *P. falciparum* for 4 years (2010, 2014, 2015 and 2016). There was a remarkable reduction of overall malaria infection during the 10 years. Malaria has been reported in all age groups, but age distribution showed that vast majority of cases were adults age 15 years and above 13,305 (73.7%). In all age groups, males were more significantly affected than females (χ^2^ = 133.0, df = 2, *P *< 0.0001). Moreover, malaria positivity rate showed a strong seasonality (χ^2^ = 777.55, df = 11, *P *< 0.0001). However, malaria cases were reported in all seasons across 10 years in the study area.

**Conclusion:**

In general, malaria positivity showed a declining trend over 10 years period in the area. However, current prevalence shows it is public health burden and needs attention for further intensification of interventions. In the study area, both *P. falciparum* and *P. vivax* co-exist and *P. vivax* is more prevalent than *P. falciparum* in almost half of the years. Therefore, malaria interventions should be strengthened in the study area.

**Electronic supplementary material:**

The online version of this article (10.1186/s12936-019-2777-z) contains supplementary material, which is available to authorized users.

## Background

Malaria remains a major public health burden globally in general, and in sub-Saharan Africa in particular, including Ethiopia. In 2016 alone, an estimated 216 million cases of malaria occurred worldwide, of which 90% were in Africa region [1]. Currently, there is a global initiative to eliminate malaria and consequently, remarkable result in malaria control has been achieved. In Ethiopia, the fight against malaria has shown a notable progress in controlling the disease over the last two decades in Ethiopia. Following this, Ethiopia has also set a goal to eliminate the disease by 2030 [2, 3]. The interventions which have been contributing to such significant decline includes; the distribution of long-lasting insecticidal nets (LLIN), indoor residual spraying (IRS); and introduction of prompt and effective treatment with artemisinin-based combination therapy (ACT) to treat uncomplicated *Plasmodium falciparum* malaria and environmental management [4–6].

In Ethiopia, two parasite species, *Plasmodium falciparum* and *Plasmodium vivax* are predominant parasite species accounting 60% and 40% of malaria cases, respectively [7, 8]. The malaria transmission in Ethiopia is seasonal with unstable transmission patterns in most areas, however, it was year-around in some lowland areas. The peak malaria transmission in Ethiopia occurs in general during the months of September to December and March to May [7, 9, 10]. The unstable transmission patterns make the country prone to cyclic epidemics occurring every 5 to 8 years [2]. However, information is scarce on malaria transmission pattern in some endemic areas of Ethiopia which is vital for evidence-based intervention by local health system.

Climatic change, which is attributed to environmental modification, determines the dynamics of malaria by limiting the survival and longevity of malaria vectors and the rate of *Plasmodium* development in the vector mosquitoes [11, 12]. Thus, the human environmental modifications such as dam construction, and extensive agricultural practices in particular area can have an impact on the trend of malaria transmission [13, 14]. In Ethiopia, there are rapid ecological changes following the development activities [14, 15]. Arjo-Didessa sugar development site is one of the largest development projects in the country. There is extensive environmental modification due to huge sugarcane plantation farm. Irrigation of sugarcane field and development in its vicinity has created a large area of malaria vector breeding habitats which may have significant impact on malaria transmission. However, malaria transmission pattern has not been yet described in and around the setting.

This study aimed at determining the malaria trend and transmission pattern for the past 10 years at the health facilities as a proxy measure for the trend of malaria at Arjo-Didessa sugar cane development site and it vicinity, which may contribute to evidence-based decision for malaria control strategies.

## Methods

### Study setting

**Fig. 1:**
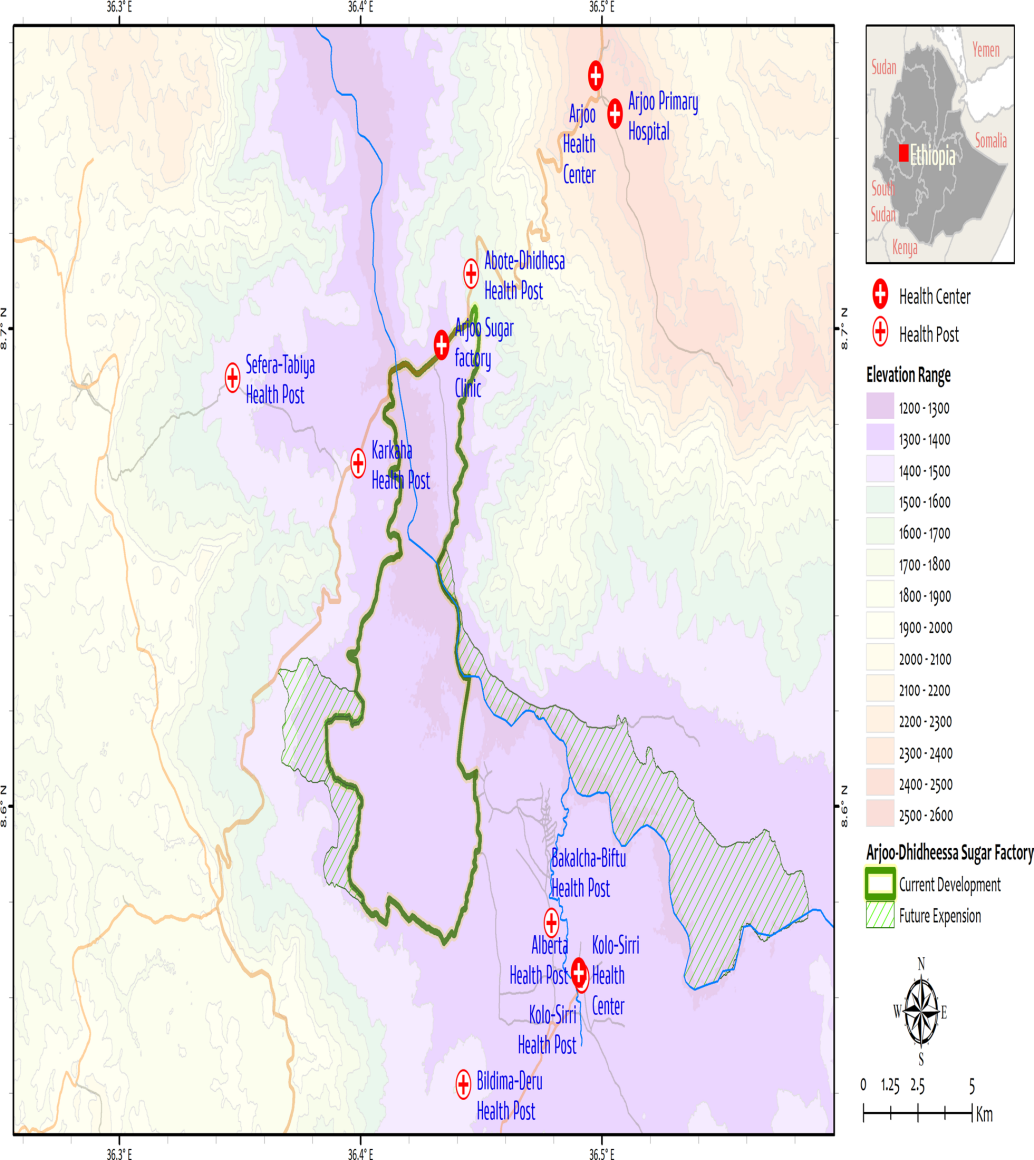
Map of the study site

The study was conducted at Arjo-Didessa sugar development site (Arjo-Didessa sugar factory) and its vicinity. This area is found between two districts, Jimma-Arjo district and Buno-Bedele district, Oromia Regional State, southwest Ethiopia (Fig. 1). The two districts have a total population of about 215, 288. Almost all of the population in the area depends on subsistence farming. The altitude of the area ranges from 1300 to 2280 m above sea level with mean annual rainfall of 1477 millimetres and the area is known to be malarious.

Historically the area is known to be wildlife sanctuary called ‘Dedessa wildlife sanctuary’, which was a forest. In 2006, a huge sugar cane plantation which supplies a state owned sugar factory has been developed. Currently, the sugar plantation is cultivated in more than 4000 hectares of land, with the planned future expansion of 80,000 ha. Eleven health facilities (health posts, health centres and a hospital) were included in this study. The health facilities included in the study are: Arjo Health Centre, Arjo Primary Hospital, Abote-Dedessa Health Post, Arjo Sugar factory Clinic, Kolo-Sirri Health Centre, Kolo-Sirri Health Post, Alberta Health Post, Bildima-Deru Health Post, Karkaha Health Post, Sefera-Tabiya Health Post and Bakalcha-Biftu Health Post. The total catchment population was estimated to be around 50,000.

### Study design and data collection

A health facility-based retrospective study was conducted by reviewing the malaria morbidity records from registers of 11 health facilities at Arjo-Didessa sugar development site and its vicinity (Fig. 1).

### Data source

In Ethiopia, malaria cases are diagnosed both clinically and using both microscope and rapid diagnostic tests (RDT) as per the national malaria diagnosis and treatment guideline [10]. Both the presumptive and confirmed cases are recorded on registration books at Primary Health Care Units (PHCU) and reported to next higher level of health management system. The study included all malaria records of those individuals who were diagnosed using microscope or RDT over the past 10 years. The timeline consideration was based on data availability and quality in the facilities.

### Data collection

All available malaria morbidity registration books were collected from the health facilities. All records of patients who presented at the health facilities and treated as malaria patients were included in the study. The data was extracted and entered into Microsoft Excel Worksheet. The parameters recorded included health facility’s name, residence, date of examination, result, gender, age, and parasite species. The cases with incomplete records of important variables and examination results were excluded from the study. Data was collected by trained laboratory technicians.

### Meteorological data collection

Meteorological records were obtained from Arjo meteorological station. Variables recorded included average monthly maximum, minimum and mean temperature, monthly cumulative precipitation and relative humidity.

### LLIN campaign data

Since there were no records of LLIN coverage in the study area for the past 10 years, national LLIN campaign data, i.e., total number of LLIN distributed nationwide, was used as a reference for malaria control interventions. LLIN campaign data was obtained from Federal Ministry of Health.

### Data analysis

Malaria infection positivity rate was calculated as number of confirmed cases over the total examined at all study health facilities. Incidence rate was calculated as cases per 1000 people per year based on current catchment population and 2007 Ethiopia census assuming constant increase in catchment population during the study period. Age was grouped as < 5 years, 5–14 years, and ≥ 15 years. Age and sex distributions were compared between 2008–2014 and 2015–2017 using χ^2^-test. Seasonality was determined by monthly positivity rate of total infections and by species. Species composition was calculated annually.

Trends, climatic and intervention effects was analysed using the following model:

$$C_{t + 1} = \alpha + \gamma t + \beta_{1} C_{t} + \beta_{2} LLIN_{t} + f\left( {CLIM} \right) + \varepsilon_{t}$$

$$f\left( {CLIM} \right) = \beta_{3} T_{max} + \beta_{4} T_{min} + \beta_{5} T_{mean} + \beta_{6} P_{recip} + \beta_{7} H_{umid}$$

where α is a constant, γ measures the trend, parameters of β measures carry-on effect (autocorrelation), LLIN effect and climatic effects, including maximum, minimum and mean temperature, precipitation and relative humidity, and ε_t_ is a random error term. Parameters were estimated using maximum likelihood estimation (MLE) and best model was selected by the Akaike information criterion (AIC). To determine whether or not there was a significant decline in incidence since 2015, analysis was carried out firstly by using data from 2008 to 2014 and then using all data.

## Results

### General characteristics and malaria parasite species

**Fig. 2:**
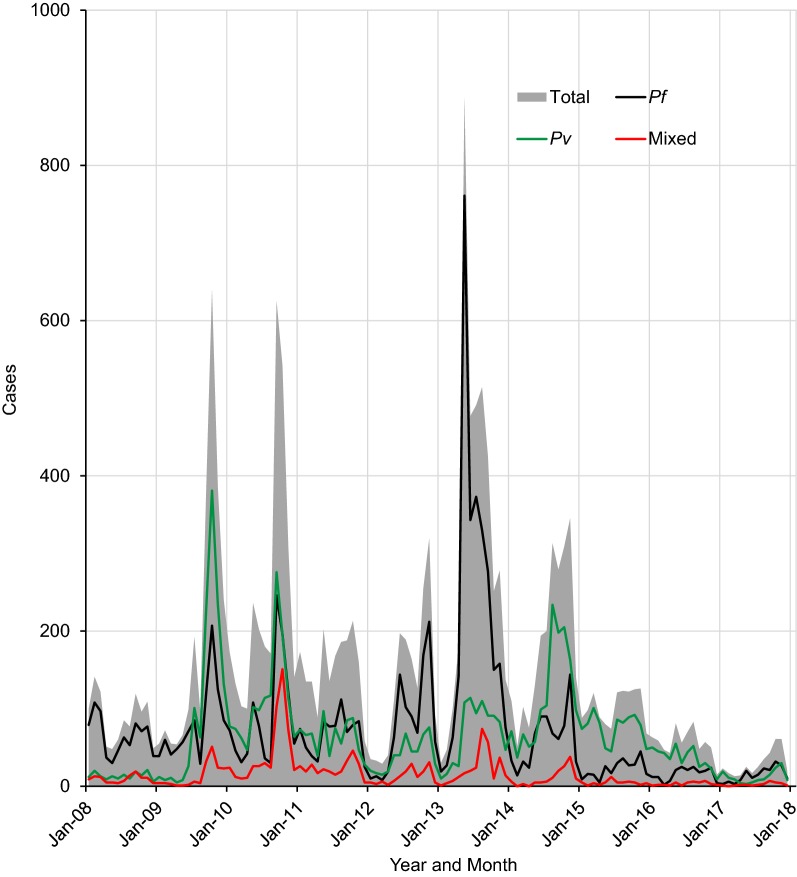
Annual trend of malaria cases at Arjo-Didessa sugar development site and its vicinity, southwest Ethiopia (2008–2017)

**Fig. 3:**
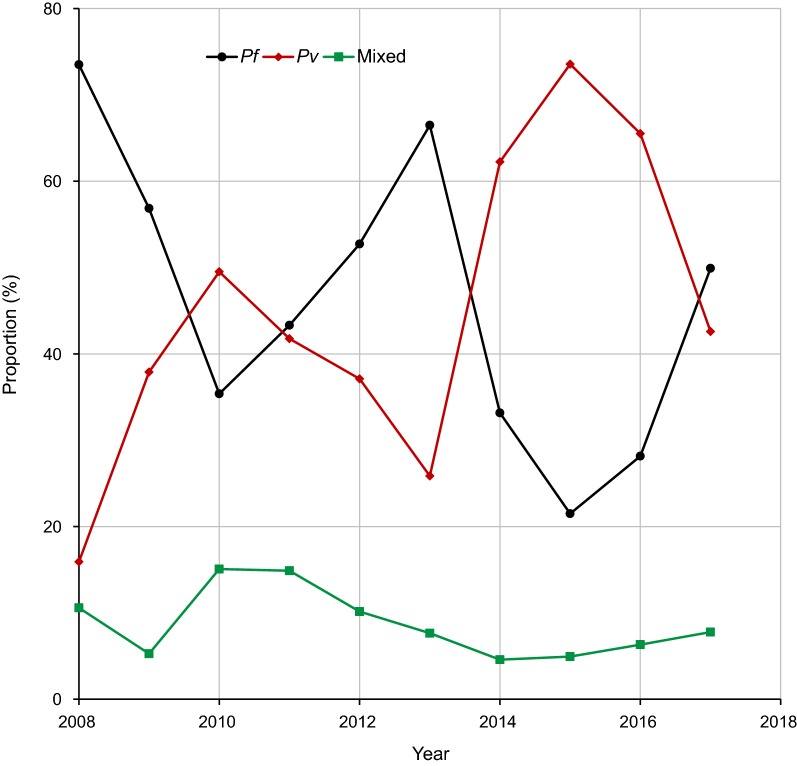
Proportion of *Plasmodium* species at Arjo-Didessa sugar development site and its vicinity, southwest Ethiopia (2008–2017)

Over a period of 10 years, 54,020 suspected malaria cases were diagnosed in the health facilities at the study area, of which 18,049 (33.4% positivity rate) were confirmed malaria cases by both microscopically and RDT. There were 3 peak years, i.e., 2009, 2010 and 2013 (Fig. 2). *Plasmodium falciparum, P. vivax*, and mixed infection accounted for 8660 (48.0%), 7649 (42.4%), and 1740 (9.6%) of malaria cases, respectively. Although overall slight predominance of *P. falciparum* over *P. vivax* was observed, *P. vivax* was dominant over *P. falciparum* for 4 years, i.e., 2010, 2014, 2015 and 2016 (Figs. 2, 3). Following the malaria peak in 2013, there was remarkable decline in malaria cases due to decline of *P. falciparum* [average of 1146 (51.6%) between 2008 and 2014 to 213 (33.2%) between 2015 and 2017] (Figs. 2, 3). Proportion of mixed infections was down from average of 231 (9.7%) between 2008 and 2014 to 41 (6.2%) between 2015 and 2017 (Figs. 2, 3).

### Age and gender distribution and their change over time

**Fig. 4:**
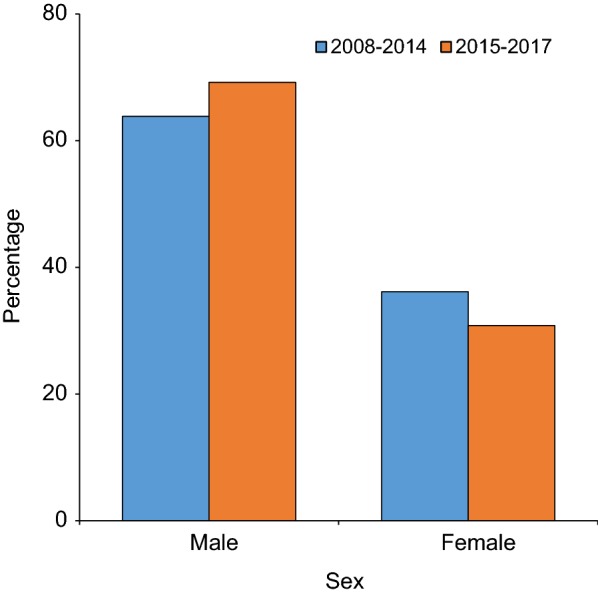
Distribution of malaria cases in different year periods across the sex at Arjo-Didessa sugar development site and its vicinity, southwest Ethiopia (2008–2017)

Of the total patients examined, 31,954 (59.2%) were males and 22,066 (40.8%) were females. Of the total malaria cases confirmed, 11,644 (64.5%) were males and 6405 (35.5%) females. Males were increasingly dominant in malaria cases, between 2008 and 2014 males accounted for 63.8% (10,082/15,792) of all cases compare to 69.2% (1562/2257) between 2015 and 2017 (χ^2^ = 68.26, d.f. = 1, *P *< 0.0001) (Fig. 4).

**Fig. 5:**
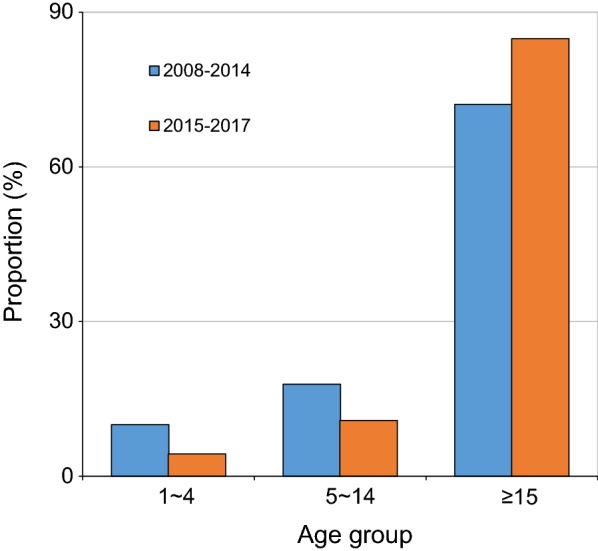
Distribution of malaria cases in different year periods across the age at Arjo-Didessa sugar development site and its vicinity, southwest Ethiopia (2008–2017)

Age distribution showed that vast majority of cases were adults age 15 years and above 13,305 (73.7%) (Fig. 5). Proportion of adult cases increased from 72.1% (11,390/15,792) between 2008 and 2014 to 84.2% (1915/2257) between 2015 and 2017 (χ^2^ = 284.15, d.f. = 1, *P *< 0.0001) (Fig. 5).

Cross examination revealed that, in all age groups, males were more affected than females, and the difference was significant (χ^2^ = 133.0, d.f. = 2, *P *< 0.0001) (Additional file 1). Cross comparison also found that *P. falciparum* was the predominant parasite in children below 15 years, however, *P. vivax* and mixed were more pronounced in adults (χ^2^ = 171.2, d.f. = 2, *P *< 0.0001) (Additional file 2).

### Seasonal variations in malaria positivity rate

**Fig. 6:**
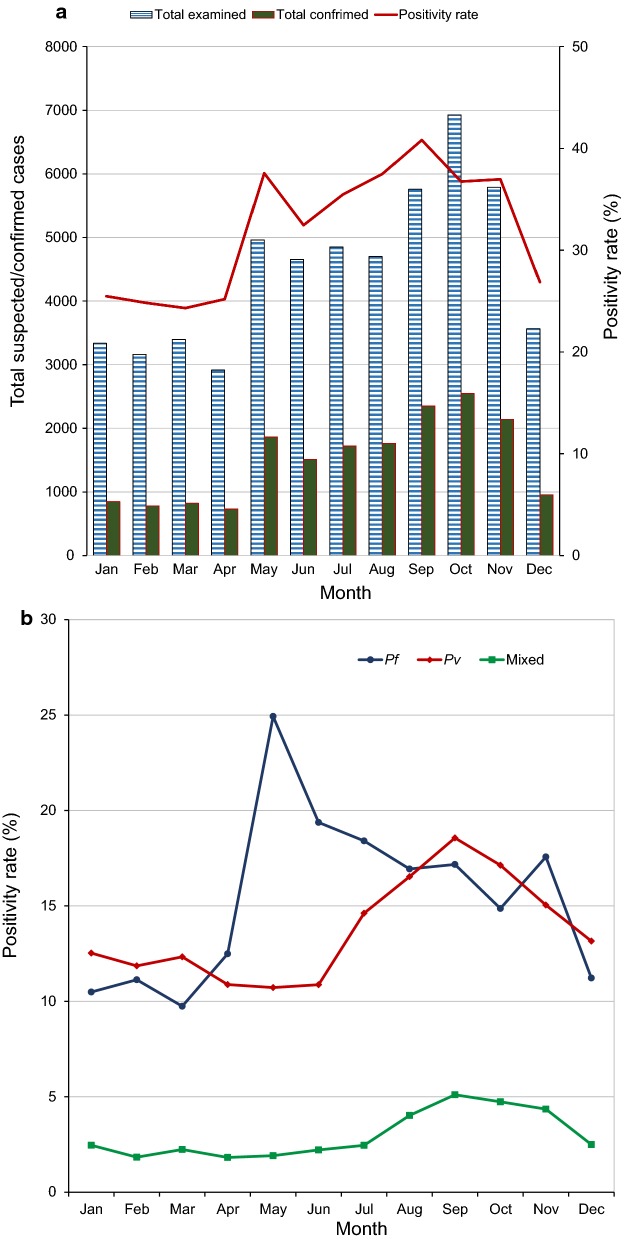
Seasonal dynamics in total malaria positivity rate (**a**) and in parasite species (**b**) at Arjo-Didessa sugar development site and its vicinity, southwest Ethiopia (2008–2017)

Examination of malaria positivity rate showed a strong seasonality (χ^2^ = 777.55, df = 11, *P *< 0.0001). The peak season started in May and ended in November with highest confirmed cases between September and November after the long-rainy season (Fig. 6a). However, there was a significant difference in peak seasons between falciparum and vivax parasites (χ^2^ = 563.52, df = 1, *P *< 0.0001) (Fig. 6b). *Plasmodium falciparum* peaked in May and dominated from May to July, *P. vivax* peaked in September, and the two species showed similar proportion from August to December (Fig. 6b).

### Trend in clinical incidence rate and climatic effect

#### Total malaria cases

Table 1 showed the results of modelling analysis for the total malaria cases (R^2^ = 0.552 adjusted R^2^ = 0.536, F_6,113_ = 34.806, P < 0.001). In addition to significant 1-month lagged carry-on (infections carried from last month) effect, 2-month lagged precipitation had significant positive impact on total cases, an increasing trend before 2013 and a decreasing trend (represented by time) after 2013 was observed (Table 1).

**Table 1:** Modelling analysis of the trend in clinical malaria incidence and climatic effect

Parasite species	Term	Estimate	t-value	Prob > |t|
Total cases	Total cases from last month	0.543	7.42	< 0.0001
	Precipitation (2-month lagged)	0.196	3.58	0.0005
	Time (month since Jan 2008)	− 0.478	− 1.90	0.0599
	(Time-61.5)*(Time-61.5)	− 0.025	− 2.80	0.0060
*P. falciparum*	*P. falciparum* cases from last month	0.530	6.73	< 0.0001
	Mixed cases 2 months ago	− 0.683	− 2.01	0.0470
	Precipitation (2-month lagged)	0.132	3.25	0.0015
	Minimum temperature (1-month lagged)	14.461	2.53	0.0128
	Time (month since Jan 2008)	− 0.494	− 2.54	0.0124
	(Time-61.5)*(Time-61.5)	− 0.016	− 2.43	0.0169
*P. vivax*	*P. vivax* cases from last month	0.836	9.50	< 0.0001
	Mixed cases from last month	− 0.855	− 2.95	0.0038
	Precipitation (2-month lagged)	0.101	3.98	0.0001
	Time (month since Jan 2008)	− 0.248	− 2.15	0.0335
	(Time-61.5)*(Time-61.5)	− 0.009	− 2.40	0.0182

#### *Plasmodium falciparum* cases

Table 1 showed the results of modelling analysis for *P. falciparum* cases (R^2^ = 0.524, adjusted R^2^ = 0.498, F_6,111_ = 20.345, P < 0.001). For *P. falciparum* cases, in addition to significant 1-month lagged carry-on effect and 2-month lagged precipitation effect, 1-month lagged minimum temperature had positive impact on clinical cases. Similar temporal trend was revealed with modelling analysis, i.e., an increasing trend before 2013 and a decreasing trend (represented by time) after 2013 (Table 1).

#### *Plasmodium vivax* cases

**Fig. 7:**
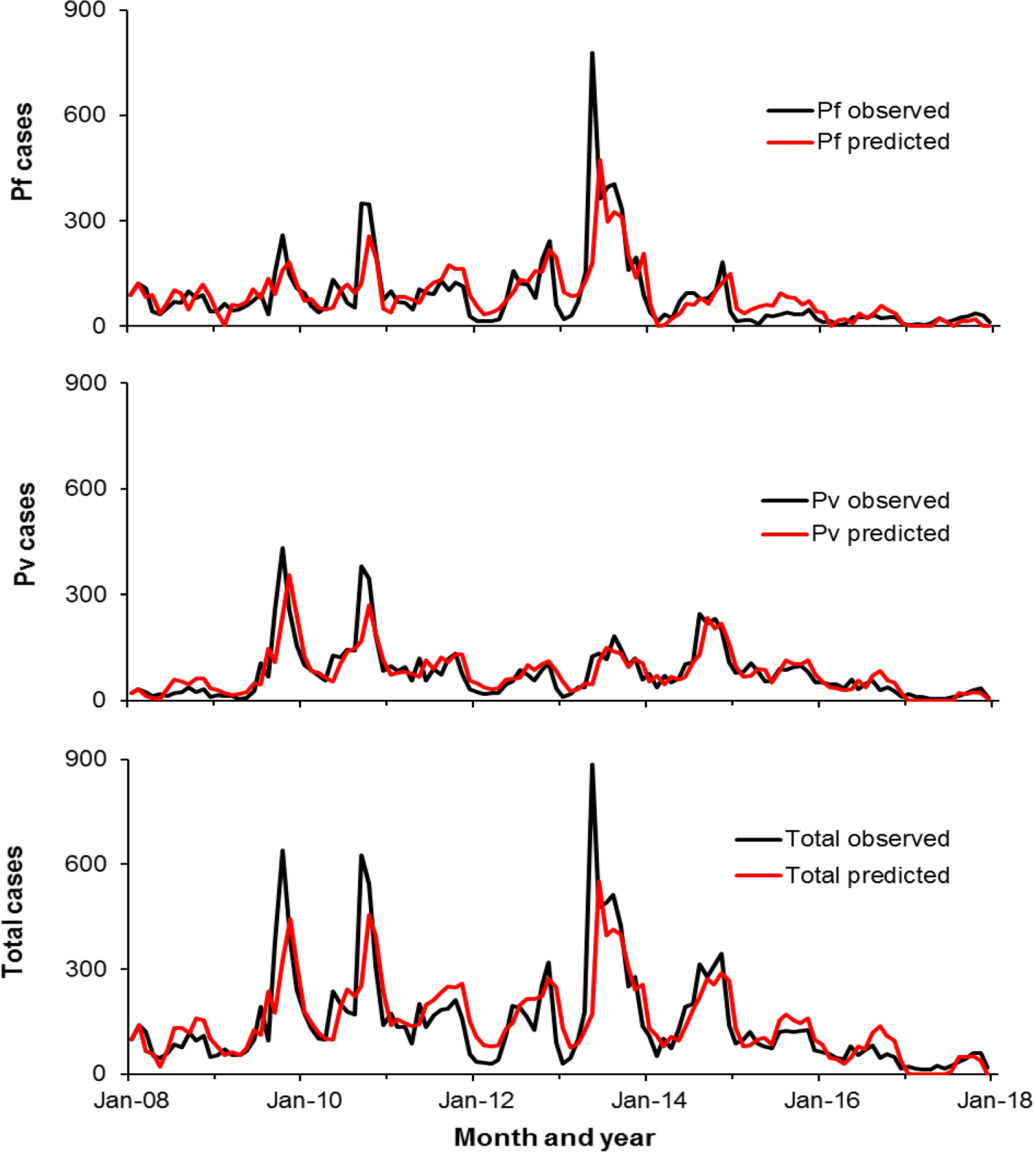
Models prediction

Table 1 showed the results of modelling analysis for *P. vivax* cases (R^2^ = 0.674, adjusted R^2^ = 0.659, F_6,111_ = 40.285, P < 0.001). For *P. vivax* cases, again 1-month lagged carry-on effect, 2-month lagged precipitation effect and similar temporal trends were significant factors (Table 1). The models predicted well of the three major peaks in 2009, 2010 and 2013 (Fig. 7).

## Discussion

The results of this study indicated that malaria was a major public health burden in the area. Over the 10 years, a 31.8% annual mean positivity rate of malaria was reported. This was lower than the findings of studies conducted at Kola Diba health centre and Adi Arkay, Northwest Ethiopia [4, 16]. The observed differences might be due to a difference in micro-eco-epidemiological settings. The variation in malaria diagnosis techniques and the skills of the laboratory personnel in detecting and identifying malaria parasites might also be one of reasons for the discrepancies. Likewise, the implementation of malaria prevention and control activities might differ from one area to another which indicates that the interventions in this area might have been stronger.

This study also revealed that malaria cases due to *P. falciparum, P. vivax* and their mixed infection accounted for 48, 42.4 and 9.6% of the cases, respectively. This is not consistent with the national malaria parasite distribution pattern in Ethiopia [17], which showed that *P. falciparum* and *P. vivax* accounting for 60 and 40% of the cases, respectively. The national figure estimation of malaria parasites indicates the average distribution in the country as whole, while this study is limited to a small malaria endemic setting in a country could have resulted in the variation of the species prevalence. Similarly, the results of present study is not consistent with the reports from other parts of the country, which revealed malaria cases due to *P. falciparum*, *P. vivax* and their mixed infection accounted for 68.9, 28.8 and 2.3% of the cases, respectively [16]. Another similar study also reported *P. falciparum* and *P. vivax* accounted for 75 and 25% of malaria morbidity, respectively [4]. This study also shows a trend in malaria parasite species shift in which *P. vivax* has become predominant over *P. falciparum* in the years 2010, 2014, 2015 and 2016. Such trend in malaria parasite prevalence shift had also been reported by another similar study [18]. It has been reported that *P. falciparum* is more prevalent and fatal malaria in Ethiopia. Because of this the intervention activities of malaria mainly focus on *falciparum* malaria, which might be a plausible reason for the observed trend shift [19]. Other possible reasons might be climate variability due to environmental modification at the area and *P. vivax* might have developed resistance for the currently available drug, chloroquine [18, 20].

The present study also revealed higher positivity rate of malaria among males (64.5%) than females (35.5%). This result is in agreement with previous local studies [4, 16, 21, 22], which had indicated that higher malaria prevalence in males than females. Age distribution showed that vast majority of cases were adults age 15 years and above followed by the age group of 5–14 years (Fig. 5). Such results, had been reported by other studies [4, 16], and other studies had also reported more susceptibility in the age range of 5–14 years [22, 23]. The possible justification for malaria affected males might be due to the fact that, the life of community is totally depends on farming and most of the time males in reproductive age group are engaged in farming activities during which they are more likely exposed to the infective mosquito bites. The proportion of adult cases increased from 72.1% between 2008 and 2014 to 84.2% between 2015 and 2017, following the decline of average malaria positivity rate from 38.2% to 17.8% between 2008 and 2014 and 2015–2017, respectively. It has been reported that at high transmission intensities, children acquire immunity rapidly and so are not susceptible to disease when they get older. On the other hand, at low transmission there is less disease among younger children, and consequently, older children acquire less immunity and remain susceptible [24–26]. Thus, the observed malaria burden among older age group might be due to dropping transmission at the area. Other potential explanations for increasing malaria cases among older children and adult could be a preferential ITN use for younger children. Recent studies support the view that adults are also emerging as a population that deserves monitoring on the bases that they are reported to be at increased risk of malaria probably due to the declining anti-malarial immunity that follows decreased exposure to parasites which could be a new challenge for elimination [25, 27]. Therefore, as control interventions could induce change in malaria epidemiology, including gender and age structure, the control strategies need to be updated to contemporary epidemiological context to be able to respond to transmission dynamics over time [28].

Over the study period, there was a declining trend of total malaria cases. This overall decline of total malaria could be attributed to the decline in *P. falciparum*, *P. vivax* and mixed infection. The possible reason for this decline of malaria might be the increased attention to scaled-up malaria control interventions by national malaria control programme (NMCP) of Ethiopia since 2004. Ethiopia launched multiple interventions for malaria prevention and control throughout the country including the study area [5, 29]. The national malaria elimination strategic plan currently recommends key intervention methods including prompt diagnosis using RDTs, ACT as first-line treatment for uncomplicated *P. falciparum* malaria, and targeting the vector using indoor residual spraying (IRS), long-lasting insecticide-treated nets (LLITNs) and environmental management [9, 17, 30]. Moreover, in the past decades, malaria control and prevention activities were intensified in the study area as it has been for other parts of the country. Community awareness creation about malaria prevention and control methods, increased accessibility of LLITNs and high coverage IRS were the major interventions employed by NMCP [30]. Overall, these efforts might have resulted in observed decline of malaria positivity rate in the study area.

In general, despite a fluctuating trend, the result showed a successive decline in malaria prevalence starting from 2013 to 2016. However, in recent years it showed slight rise which indicates that the area needs attention to intensify the existing interventions to enhance malaria elimination efforts.

Despite significant seasonality, in the study area, malaria cases were reported in all seasons over 10 years. The peak season of total positivity rate started in May and ended in November with highest confirmed cases between September and November after the long-rainy season. This finding is consistent with reports from other parts of Ethiopia [4, 18, 22], which had showed the highest peak of malaria in the months of September, October and November. In Ethiopia, the peak malaria transmission occurs between September and December following the June to September long rains [2, 7]. Some localities also experience persistent malaria, because the environmental and climatological situations permit the continuous availability of vector breeding sites [5]. In the study area, there is sugar development site with mega sugar cane plantation. This farming site has irrigation system which could increase the proliferation of mosquitoes breeding habitats. Such extensive irrigation activities can modify the environment in a favour of malaria transmission [20, 31–33]. Thus, to have better understanding of vector ecology and malaria transmission intensity in the study area, a mega research project is under way, to which this study is a part.

In this particular study, trends of malaria was measured by analysing a 10-year secondary data obtained from health facilities which might have some limitation like any other secondary data.

## Conclusion

In conclusion, the malaria positivity rate is declining. The declining trend of the overall positivity rate could be due to the decline of *P. falciparum*, *P. vivax* and mixed infections. However, malaria still remains a public health burden in the area, which needs attention and further intensified interventions.

This study also revealed the peculiar trend of malaria species, with a shift in predominance from *P. falciparum* to *P. vivax* in 4 years. The age group of 15 years and above was more affected, followed by the 5–14 years. In all age groups, males were more affected than females. Although there was significant seasonal variation, malaria cases were reported in all seasons across the 10 years in the area. Therefore, malaria interventions should be strengthened to sustain control and move towards elimination in such project corridors.

## Additional files

**Additional file 1.** Malaria cases by gender and age group at Arjo-Didessa sugar development site and its vicinity, southwest Ethiopia (2008–2017).

**Additional file 2.** Distribution of Plasmodium species by age group at Arjo-Didessa sugar development site and its vicinity, southwest Ethiopia (2008–2017).

## Abbreviations

MoH: Ministry of Health
SPSS: Statistical Package for the Social Science
ACT: artemisinin-based combination therapy
IRS: indoor residual spray
LLINs: long-lasting insecticidal nets
ITNs: insecticide-treated nets
NMCP: National Malaria Control Programme
RDT: rapid diagnostic test
