# Correction to: Ten years malaria trend at Arjo-Didessa sugar development site and its vicinity, Southwest Ethiopia: a retrospective study

**DOI:** 10.1186/s12936-019-2820-0

**Published:** 2019-05-28

**Authors:** Dawit Hawaria, Hallelujah Getachew, Guofa Zhou, Assalif Demissew, Kasahun Habitamu, Beka Raya, Ming-Chieh Lee, Delenasaw Yewhalaw, Guiyun Yan

**Affiliations:** 1 Yirgalem Hospital Medical College, Yirgalem, Ethiopia; 2 Department of Medical Laboratory Technology, Arbaminch College of Health Sciences, Arbaminch, Ethiopia; 3 Program in Public Health, University of California at Irvine, Irvine, CA 92697 USA; 4 Department of Medical Laboratory Sciences, College of Medicine and Health Sciences, Ambo University, Ambo, Ethiopia; 5 Department of Medical Laboratory Sciences, College of Medicine and Health Science, Kotebe Metropolitan University, Addis Ababa, Ethiopia; 6 Department of Medical Laboratory Sciences, Institute of Health, Jimma University, Jimma, Ethiopia; 7 Tropical and Infectious Diseases Research Center (TIDRC), Jimma University, Jimma, Ethiopia

## Correction to: Malar J (2019) 18:145 10.1186/s12936-019-2777-z

Following publication of the original article [1], it came to the authors’ attention that unfortunately the last name of one of the authors is spelled incorrectly in the published article.

The author name ‘Guofa Zhou’ was submitted (incorrectly) as ‘Guofa Zhong’ and shows as such in the published article.

Please find the corrected author list in this correction article.

The authors apologize for this error.
