# Supplementary figures and images for: Ten years malaria trend at Arjo-Didessa sugar development site and its vicinity, Southwest Ethiopia: a retrospective study
Source: Malar J. 2019 Apr 24;18:145. doi: 10.1186/s12936-019-2777-z (PMC6480840; doi:10.1186/s12936-019-2777-z)

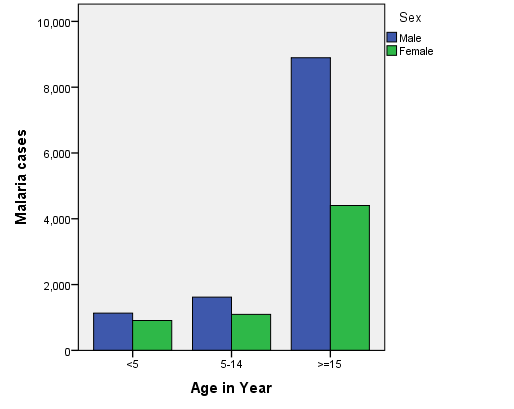

Supplement: Supplementary file 1 — Additional file 1. Malaria cases by gender and age group at Arjo-Didessa sugar development site and its vicinity, southwest Ethiopia (2008–2017). [file 12936_2019_2777_MOESM1_ESM.docx]

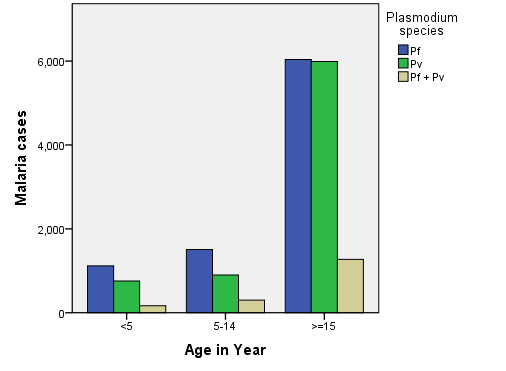

Supplement: Supplementary file 2 — Additional file 2. Distribution of Plasmodium species by age group at Arjo-Didessa sugar development site and its vicinity, southwest Ethiopia (2008–2017). [file 12936_2019_2777_MOESM2_ESM.docx]
